# Obesity and Atrial Fibrillation: A Narrative Review

**DOI:** 10.7759/cureus.31205

**Published:** 2022-11-07

**Authors:** Vasu Gupta, Jaskaran S Munjal, Poonamdeep Jhajj, Shinedeep Jhajj, Rohit Jain

**Affiliations:** 1 Internal Medicine, Dayanand Medical College and Hospital, Ludhiana, IND; 2 Internal Medicine, Shri Ram Murti Smarak Institute of Medical Sciences, Bareilly, IND; 3 Internal Medicine, Avalon University School of Medicine, Willemstad, CUW; 4 Internal Medicine, Avalon University School of Medicine, ​​Willemstad, CUW

**Keywords:** stroke, heart structural remodeling, weight loss, obesity, atrial fibrillation (af)

## Abstract

Obesity and atrial fibrillation in the elderly population both present as significant health issues worldwide indirectly. Cases of atrial fibrillation are also rising worldwide, making it the most common type of cardiac arrhythmia. There are a variety of risk factors for atrial fibrillation. Modifiable factors include smoking, hypertension, diabetes mellitus, sedentary lifestyle, obesity, and non-modifiable include genetic predisposition. Obesity is a well-known risk factor for multiple systemic conditions like metabolic syndrome, diabetes mellitus type 2, sleep-related disorders, etc. In addition, it contributes directly to the development of various cardiovascular disorders like hypertension, myocardial infarction, and, more notably, atrial fibrillation. There are multiple mechanisms by which obesity in adults increases the risk of atrial fibrillation. Some of them are systemic inflammation caused by the increased secretion of cytokines by adipocytes, neuro-hormonal disturbances and heart structure remodeling, and weight-loss strategies have shown improvements in patients suffering from atrial fibrillation.

## Introduction and background

Atrial fibrillation, a commonly occurring cardiac arrhythmia, affects three to six million general population in the United States [1]. It accounts for more than 454,000 hospitalizations and about 158,000 deaths in the United States each year [2]. It is projected to affect six to 16 million people in the United States by 2050 [2]. From 1997 to 2017, the incidence and prevalence of atrial fibrillation have increased by 31% and 33%, respectively [3]. It is also the most common sustained arrhythmia causing various complications like stroke, congestive heart failure, and death [4]. The patient usually presents with complaints of rapid and uneven heart rate, fatigue, and shortness of breath [5]. Almost 30% of the world population can be classified as obese, defined as a BMI of more than 30 kg/m^2^. Caballero suggests that more than two billion people suffer from this growing epidemic [6]. Increasing adipose tissue due to obesity is a risk factor for most cardiovascular pathologies like congestive heart failure, hypertension, and atrial fibrillation [7], with obesity causing a 50% increase in atrial fibrillation [8]. Obesity is also associated with diabetes mellitus type 2, dyslipidemia, and sleep-related breathing disorders [9]. A population-based cohort study consisting of 51,646 individuals showed a causal association between atrial fibrillation and body mass index (BMI) [10]. In another cohort involving 34,309 women, BMI and risk of atrial fibrillation had a linear relationship with a 4.7% rise with each point increase in BMI [11]. Studies have also reported a paradoxical phenomenon where overweight and obese people have a better prognosis of cardiovascular events than non-obese population [9]. Various factors account for this increased risk seen in obese patients. It has been shown that obesity leads to structural remodeling of the heart by altering the hemodynamics, which is left atrial enlargement, which is known to cause atrial fibrillation [12]. Animal study models have also demonstrated that obesity-driven inflammatory markers increase the susceptibility of atrial fibrillation and are a crucial driver of the process [13]. The relationship between high BMI and the incidence of atrial fibrillation is also evident because weight loss and lifestyle modifications in obese patients are beneficial and an important part of management in patients with persistent atrial fibrillation [14].

## Review

Pathophysiology

Obesity causes hemodynamic changes that alter cardiac morphology and physiology [15,16]. It is associated with various systemic disorders such as metabolic syndrome, hypertension, diabetes, obesity hypoventilation syndrome, obstructive sleep apnea (OSA), and dyslipidemia, all of which can increase the risk of atrial fibrillation [17]. Obesity causes a high output state due to an increase in total blood volume, which causes an increased cardiac output and filling pressures leading to left ventricular enlargement [18]. Obesity hypoventilation syndrome and OSA can also increase pulmonary artery pressures leading to right ventricular hypertrophy [18]. This, in conjunction with hypertension, causes concentric hypertrophy and left ventricular diastolic dysfunction, eventually leading to systolic dysfunction [19]. This leads to a significant increase in left atrium pressure, an increase in left atrial wall thickness, left atrial enlargement, insufficient left atrial emptying, dysfunction of the left atrium, and ultimately atrial fibrillation [20]. However, even after adjusting for age and sex, studies have found obesity a more potent predictor of left atrial remodeling than hypertension [16]. A pre-clinical study done by McCauley et al. demonstrated that obesity was associated with an increased expression of cardiac sodium and potassium ion channels and atrial fibrosis which may play a role in inducible atrial fibrillation and mitochondrial antioxidant therapy reduces the incidence of atrial fibrillation and reverses atrial fibrosis [21].

Gene expression of all renin-angiotensin-aldosterone system (RAAS) components, including aldosterone, increases in obese patients [22]. Aldosterone causes atrial fibrosis and conduction pathway disturbances, a risk factor for atrial fibrillation [23]. The Framingham Heart Study, a 38-year-long prospective cohort study, revealed that diabetes also predisposes the person to atrial fibrillation [24,25]. Diabetes, in conjunction with obesity, also causes myocardial fibrosis, induces structural changes in the atria by oxidation, and increases the concentration of advanced glycation end-products [26]. It also causes interatrial conduction delay, damaging atrial excitation-contraction coupling, and further contributes to atrial fibrillation [27].

Epicardial adipose tissue (EAT) is adipose tissue surrounding the heart, located between the myocardium and the visceral pericardium [28], and meta-analysis suggests that it is more predominant in patients with atrial fibrillation than in healthy controls [29]. EAT is a better predictor of atrial fibrillation than other measures of adiposity, such as BMI, waist circumference, waist-to-hip ratio, and intrathoracic fat [30]. Studies have shown that EAT volume correlates with serum c-reactive protein (CRP) in those with recurrent atrial fibrillation suggesting inflammation as the mediator between EAT and the risk of cardiac arrhythmias [31]. Apart from providing a fat cushion to the myocardium, EAT secretes cytokines such as vascular endothelial growth factor-1, interleukins, transforming growth factor-β, metalloproteinase 1-13, tissue necrosis factor-α, CRP, monocyte chemoattractant protein-1, and adipokines such as adiponectin, leptin, resistin, visfatin, and omentin, all of which are involved in atrial remodeling [32]. In long-term obesity, there is greater EAT and, therefore, a greater risk of developing fibrosis, which provides a substrate for reentry circuits in the myocardium [33]. Apart from causing fibrosis, EAT infiltration may also separate myocardial fibers, thus disrupting the normal conduction pathway [34].

Until now, it has been established how obesity is associated with AF. However, a 12-month longitudinal observational study done by Rodriguez-Reyes et al. on patients with documented atrial fibrillation concludes that mortality in these patients was inversely related to high BMI [35]. Meta-analysis also shows a decrease in all-cause mortality in elderly obese patients compared to non-obese patients [36]. This phenomenon is called the obesity paradox which may be because of a confounding bias due to age, more significant metabolic reserves in obese patients, cardiorespiratory fitness, increased muscle mass, and selection bias [16].

Diagnosis and treatment

Diagnosis

Patients with atrial fibrillation can present with mild or no symptoms. Atrial fibrillation can also be found in patients with other conditions such as congestive heart failure, myocardial infarction, stroke, or shock. A prospective study showed that coronary artery disease was seen in 70% of the patients suffering from atrial fibrillation and hence it is also vital to rule out any co-existent coronary artery disease [37]. A detailed history should be taken regarding any comorbidities and previous history of atrial fibrillation and a thorough examination should be performed. ECG is essential in diagnosing atrial fibrillation [38]. Where an abnormal ECG suggestive of atrial fibrillation can confirm the diagnosis, a regular reading does not entirely rule out the presence of atrial fibrillation because it may not capture paroxysmal arrhythmia. Thus, a Holter monitor (24-hour recording) or event monitor (7- to 30-day recording) may be used to diagnose such cases [39].

Initial laboratory work to evaluate the cause of atrial fibrillation includes a complete blood count, a basic metabolic panel for electrolyte abnormalities, and thyroid function tests to assess for hyperthyroidism, blood alcohol, and other illicit drug levels. It is also important to assess cardiac biomarkers and B-type natriuretic peptides to determine any underlying cardiac disease such as myocardial ischemia, myocardial infarction, or heart failure. Transthoracic echocardiography should be done to rule out structural heart diseases, like atrial septal defects and valvular diseases, and a chest x-ray to rule out pulmonary diseases.

Treatment

Management includes lifestyle modification, risk factor management, weight loss, rate and rhythm control, and anticoagulation. Studies have shown the positive effect of weight loss and risk factor management on atrial fibrillation progression and recurrence [40]. For example, the supervised obesity reduction trial for AF ablation (SORT-AF) demonstrated that well-structured weight loss programs were beneficial for obese patients with atrial fibrillation who underwent ablation by decreasing the recurrences in adult population suffering from persistent atrial fibrillation [14]. A randomized control trial of 150 Australian adults reported a decrease in atrial fibrillation disease burden, symptom severity, and disease recurrence in the intervention group undergoing weight reduction compared to the control group [41]. Furthermore, sustained weight control and metabolic bariatric surgery have shown a reduced atrial fibrillation disease burden in the long term [42,43].

A patient’s high BMI can change the pharmacodynamics and pharmacokinetics of warfarin. Anticoagulation is one of the pillars of atrial fibrillation management to minimize thromboembolic risk [17]. Patients with high BMI (more than 40 kg/m^2^) have significantly higher warfarin requirements than patients with lower BMI. Direct oral anticoagulants would be an alternative to warfarin, but minimal clinical trial data exists in patients with high BMI. A study by Martin et al. in 2016 suggests the avoidance of direct oral anticoagulants (DOACs) in morbidly obese patients due to limited clinical data [44]. Some studies indicate that direct oral anticoagulants provide consistent efficacy and safety compared with warfarin across all categories of BMI. The International Society on Thrombosis and Haemostasis does not support the use of direct oral anticoagulants in patients with a BMI of >40 kg/m^2^ or >120 kg/m^2^ [45].

Another component of atrial fibrillation management is rhythm control with direct current cardioversion (DCCV). This facilitates quick evaluations of symptoms and assesses cardiac dimensions and function changes while in sinus rhythm. Patients with higher BMI have shown lower success rates with cardioversion [17]. Due to adiposity, the lower success rate likely results from reduced energy delivery to the heart [15].

## Conclusions

Atrial fibrillation is a common arrhythmia and a significant cause of cardiovascular mortality. The prevalence of obesity is also rising, and it has been classified as an epidemic by various studies. Obesity has long been known to increase the risk of various cardiovascular diseases, and its role in causing atrial fibrillation is undisputed. There are multiple mechanisms, like the structural remodeling of the heart, epicardial adipose tissue, and inflammatory markers secreted by the fatty tissues which may lead to atrial fibrillation in obese adult population. The causal relationship is also evident because the studies done over the years have reported a positive role of weight loss in treating and preventing atrial fibrillation in obese patients. Apart from weight loss and lifestyle modification, rate and rhythm control complete the management of atrial fibrillation. Our study is limited only to the adult population and is a narrative review that does not provide any statistically significant association between obesity and atrial fibrillation. More extensive research is needed to strengthen this association with strategies to prevent obesity and associated atrial fibrillation.
